# Feasibility of the Assessment of Cholesterol Crystals in Human Macrophages Using Micro Optical Coherence Tomography

**DOI:** 10.1371/journal.pone.0102669

**Published:** 2014-07-21

**Authors:** Manabu Kashiwagi, Linbo Liu, Kengyeh K. Chu, Chen-Hsin Sun, Atsushi Tanaka, Joseph A. Gardecki, Guillermo J. Tearney

**Affiliations:** 1 Harvard Medical School and Wellman Center for Photomedicine, Massachusetts General Hospital, Boston, Massachusetts, United States of America; 2 School of Electrical & Electronic Engineering and School of Chemical & Biomedical Engineering, Nanyang Technological University, Singapore, Singapore; 3 Harvard-MIT Division of Health Sciences and Technology, Cambridge, Massachusetts, United States of America; 4 Department of Pathology, Harvard Medical School and Massachusetts General Hospital, Boston, Massachusetts, United States of America; Maastricht University, Netherlands

## Abstract

The presence of cholesterol crystals is a hallmark of atherosclerosis, but until recently, such crystals have been considered to be passive components of necrotic plaque cores. Recent studies have demonstrated that phagocytosis of cholesterol crystals by macrophages may actively precipitate plaque progression via an inflammatory pathway, emphasizing the need for methods to study the interaction between macrophages and crystalline cholesterol. In this study, we demonstrate the feasibility of detecting cholesterol in macrophages *in situ* using Micro-Optical Coherence Tomography (µOCT), an imaging modality we have recently developed with 1-µm resolution. Macrophages containing cholesterol crystals frequently demonstrated highly scattering constituents in their cytoplasm on µOCT imaging, and µOCT was able to evaluate cholesterol crystals in cultured macrophage cells. Our results suggest that µOCT may be useful for the detection and characterization of inflammatory activity associated with cholesterol crystals in the coronary artery.

## Introduction

Cholesterol crystals are generally considered hallmarks of atherosclerosis, though their roles have long been thought to be passive elements of necrotic cores [1], [2], imparting mechanical stability and stiffness to atherosclerotic lesions [3]. Recent studies have indicated that macrophage phagocytosis of cholesterol crystals may precipitate plaque progression by stimulating the nucleotide-binding domain and leucine-rich repeat containing proteins 3 (NLRP3) inflammasome pathway [4], [5]. After cholesterol crystals are phagocytosed by macrophages, lysosomal destabilization and leakage of cathepsin B into the cytoplasm follow, where the enzyme indirectly activates the NLRP3 inflammasome. These findings have heightened interest in the interaction between macrophages and cholesterol crystals and have created a demand for an imaging modality capable of visualizing macrophage phagocytosis of cholesterol crystals as a tool for evaluating inflammatory activity in atherosclerosis. Cholesterol monohydrate crystals are birefringent and change the polarization of transmitted light. For this reason, polarization microscopy is considered a gold standard optical microscopy technique for visualization of cholesterol crystals in cells *in vitro*. In histologic slides of human atherosclerotic plaque, cholesterol crystals appear as oblong clefts that represent the voids left behind following histopathologic processing. Techniques for assessment of crystalline cholesterol *in situ* however are lacking. [6], [7].

Optical coherence tomography (OCT) is the highest resolution intracoronary imaging modality available currently, which provides an axial resolution of 10–20 µm and a transverse resolution of 20–40 µm [8]–[11]. *In vivo* OCT studies have been shown to elucidate the mechanism of acute coronary syndrome and atherosclerosis [12]–[14] and reports have suggested that OCT may be capable of identifying macrophage accumulations and large, extracellular cholesterol crystal plates [15]. Recently, we have developed new OCT technology termed Micro-OCT (µOCT), which exhibits ten-fold improvement in resolution along every spatial direction compared to conventional OCT. µOCT has shown an improved capability to visualize subcellular features of the human coronary artery compared with conventional OCT, including the visualization of individual macrophages and detailed morphology of extracellular cholesterol crystals [16].

In this study, we aimed to determine the feasibility of using µOCT to evaluate and quantify macrophages and cholesterol crystals with a particular emphasis on the macrophage-cholesterol crystal interaction by imaging cultured human macrophages and cadaver human coronary arteries.

## Methods

### Ethics Statement

The Institutional Review Board at the Massachusetts General Hospital approved the studies using human blood (IRB #2011P002726) and human arterial tissue (IRB #2004P000578). The written informed consents from donors were obtained for human blood draw and attached into the manuscript. The human arterial tissues have been described in the previous publication [16].

### µOCT system

**Figure 1:**
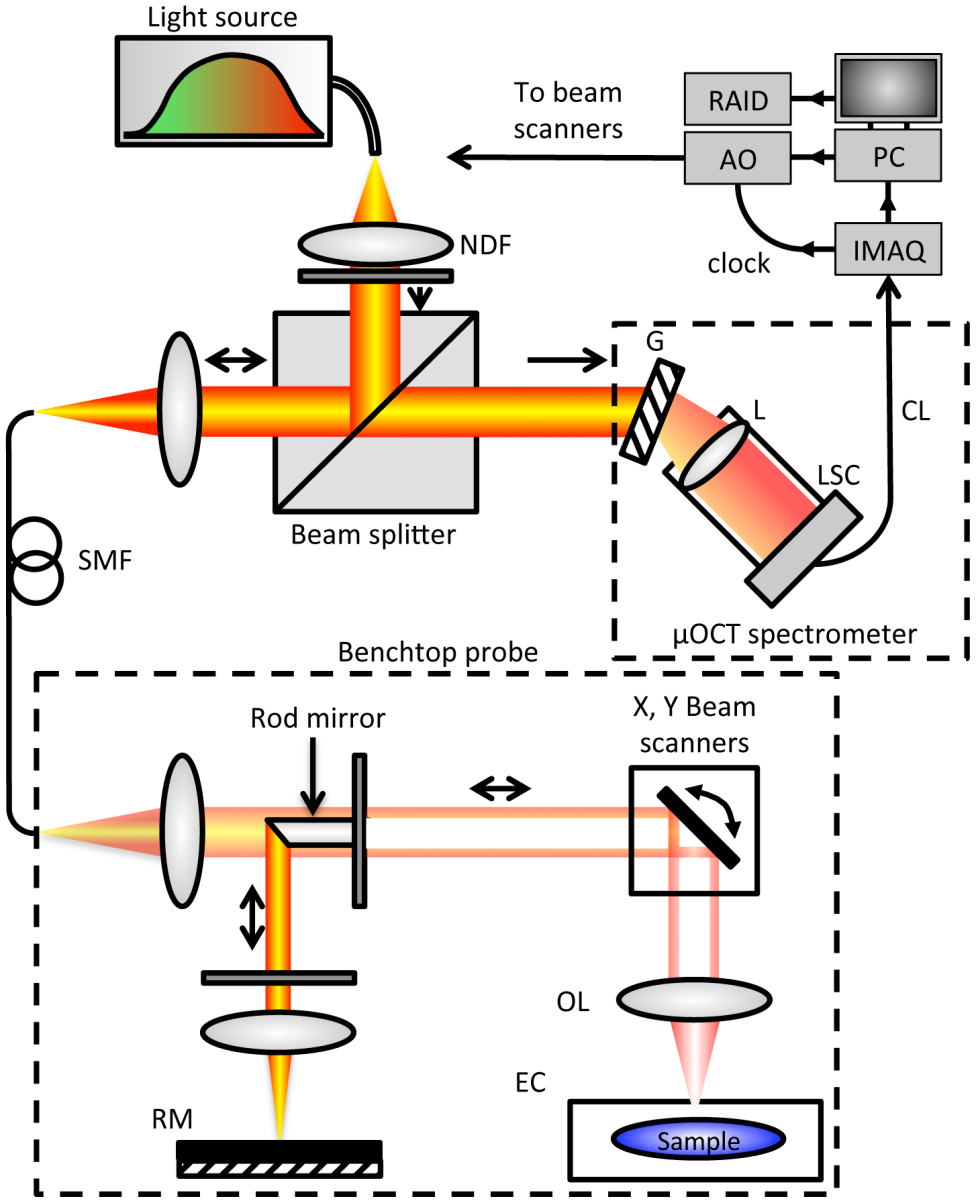
µOCT instrumentation schematic. System diagram. RM: reference mirror. OL: objective lens. EC: environmental chamber. AO: analog output board. G: grating. IMAQ: image acquisition board. L: camera lens. LSC: line scan camera. SMF: single mode fiber. PC: personal computer. RAID: redundant array of independent disks. CL: Camera Link cable.

OCT measures the electric field amplitude of light that is elastically scattered from within tissue in three dimensions. Depth or axial (*z*) ranging is achieved by interferometric measurement of the optical delay of light returned from the sample. µOCT, as implemented here, is based on a form of OCT known as spectral-domain OCT with several key improvements that yield high resolution in both lateral and axial directions (Figure 1) [17]. A super-continuum source (SuperK OCT Extreme, NKT Photonics, Birkerød, Denmark) provides high-bandwidth (600 nm to 1800 nm) short coherence length light, of which the spectral range from 650 nm to 950 nm is utilized by µOCT, resulting in high axial resolution (1.3 µm in air). Light is delivered to and collected from the interferometer optics via SM600 fiber (Thorlabs, Newton NJ). To achieve an acceptable balance between high lateral resolution (2 µm) and sufficient depth of field (0.2 mm), µOCT replaces the beamsplitter element typically used in OCT with a 45 degree rod mirror, which redirects the center portion of the illumination into the reference arm and introduces a circular obscuration in the center of the sample beam. The rod mirror obscures a region corresponding to a numeral aperture (NA) of approximately 0.06, in comparison to the 0.12 total beam NA. The annular geometry of the sample beam both enhances lateral resolution and improves depth of field [18], but does not sacrifice as much sensitivity and mitigates the presence of sidelobe artifacts compared to a fully Bessel-beam configuration. The total power incident on the sample is 10 mW. Custom software was created to control the galvanometer scanning motors while acquiring spectral data from the line camera (Sprint spL4096-140k, Basler AG, Ahrensburg, Germany).

### Macrophage Cell culture

Macrophage culture was performed as described in a previous report [19]. Human peripheral blood mononuclear cells were isolated from healthy donors by density gradient centrifugation with Histopaque-1077 (Sigma-Aldrich, Saint Louis, Missouri) and washed 3 times in Dulbecco's phosphate-buffered saline (Life Technologies, Carlsbad, California). Cells were suspended in RPMI 1640 (supplemented with 10% human serum, 40 µg/ml gentamicin, and 2 mM glutamine), and incubated for 4 hours at 37°C with 5% CO_2_. Nonadherent cells were discarded, and adherent monocytes were maintained in RPMI 1640 for 7 days.

Foam cell differentiation in resting macrophages occurred after 7 days in culture with human oxidized low-density lipoprotein (LDL). Human oxidized LDL (Intracel Resources) was added at day 8, and after 7 days, foam cells were incubated with cholesterol monohydrate crystals for 24 hours. Synthetic cholesterol (Sigma-Aldrich) was solubilized in hot acetone and crystallized by cooling [4]. After six cycles of recrystallization, the final crystallization was performed in the presence of 10% endotoxin-free water to obtain hydrated cholesterol crystals, which were confirmed by measuring angles of crystals on µOCT (79.81±2.98 (acute) and 102.13±3.89 (obtuse) degrees) [20]. Crystal size was varied with a micro-tube tissue grinder (Sigma-Aldrich). At day 16, cultured cells were examined by polarization microscopy and µOCT. µOCT imaging was performed on the cells with illumination originating from below.

### Human tissue specimens

We examined 45 human coronary arterial specimens with macrophage cells from grossly diseased arterial segments. Coronary arteries were obtained from freshly explanted human hearts provided by Capital Bioscience (Rockville, Maryland; http://www.capitalbiosciences.com/). Explanted hearts were harvested from organ donors after the cessation of vital signs, perfused with UW transplant solution and shipped on ice within a 24 hour postmortem interval. The major coronary arteries from the heart were prosected and opened longitudinally. Regions of interest were identified by gross visual inspection and optical frequency domain imaging (OFDI), a form of OCT well established for intravascular imaging [21]. Before µOCT imaging, specimens were immersed in a thin layer of phosphate buffered saline (PBS; Sigma-Aldrich) at 25°C. µOCT images were acquired from the luminal surface. The time between death and µOCT imaging did not exceed 48 hours.

### µOCT image analysis

**Figure 2:**
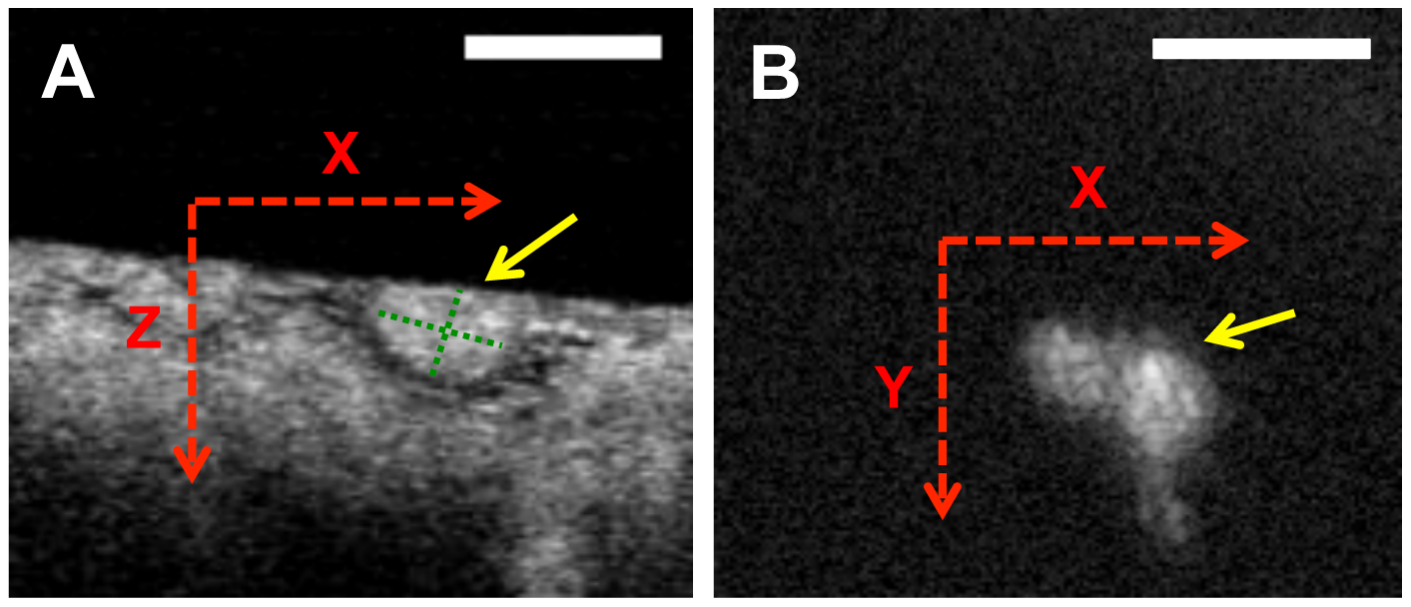
Volumetric analysis of macrophage cell and cholesterol crystal by µOCT. Cross-sectional µOCT images of macrophage cell in coronary artery (A) and cultured macrophage cell with cholesterol crystal (B). Long and short axes (green dotted lines) of macrophage were measured at the slice with biggest cross-sectional cell area. Each 2-D area of macrophage cell and cholesterol crystals was measured. Scale bars = 50 µm.

Figure 2-A shows cross-sectional µOCT image of a macrophages in coronary artery. In the coronary artery, macrophages are characterized as highly scattering, round or ellipsoidal structures that are clearly delineated from other coronary artery components (Figure 2-A). Cross-sectional measurements of macrophage areas were manually performed by ImageJ. Long and short axes of each macrophage were measured at the slice with biggest cross-sectional cell area. The volume was determined by calculating the sum of all cross-sectional areas multiplied by the slice thickness (Simpson's rule). Cholesterol crystals within cultured macrophage cells were defined as highly scattering inclusions (Figure 2-B). Intracellular crystal volumes were measured using the same technique as macrophage volume.

### Statistical analysis

Statistical analysis was performed using SPSS software for Windows version 11.0 (SPSS Inc., Chicago, Illinois). Results are expressed as medians and interquartile ranges. Qualitative data are presented as numbers and percentages satisfying the given criteria. Differences between two groups were tested by Fisher's exact test for categorical variables. Pearson correlation coefficient was used to analyze the correlation between 2 parameters. Values of p<0.05 were considered significant.

## Results

### Imaging cultured macrophages with intracellular cholesterol crystals

**Figure 3:**
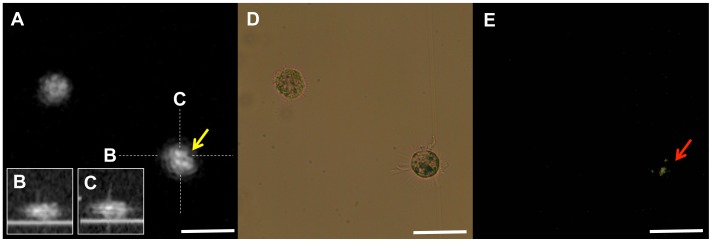
Representative image of cultured macrophage cell with cholesterol crystal. A. Representative image of a macrophage with a cholesterol crystal. The macrophage on the right demonstrated highly scattering constituents inside its cytoplasm (yellow arrow). B, C. The Cross sectional images of the macrophage on the right. D, E. Polarization microscopy confirmed that the inclusions are cholesterol crystals (red arrow). Scale bars = 50 µm.

**Figure 4:**
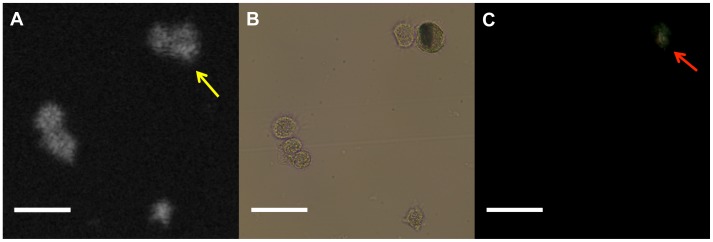
Discrepancy image of cultured macrophage cell with cholesterol crystal. A. Discrepancy image of macrophage with cholesterol crystal. Two macrophages determined to contain cholesterol crystals by polarized light microscopy (B, C red arrow) did not show definitive evidence of crystals on the µOCT image (yellow arrow). Scale bars = 50 µm

**Figure 5:**
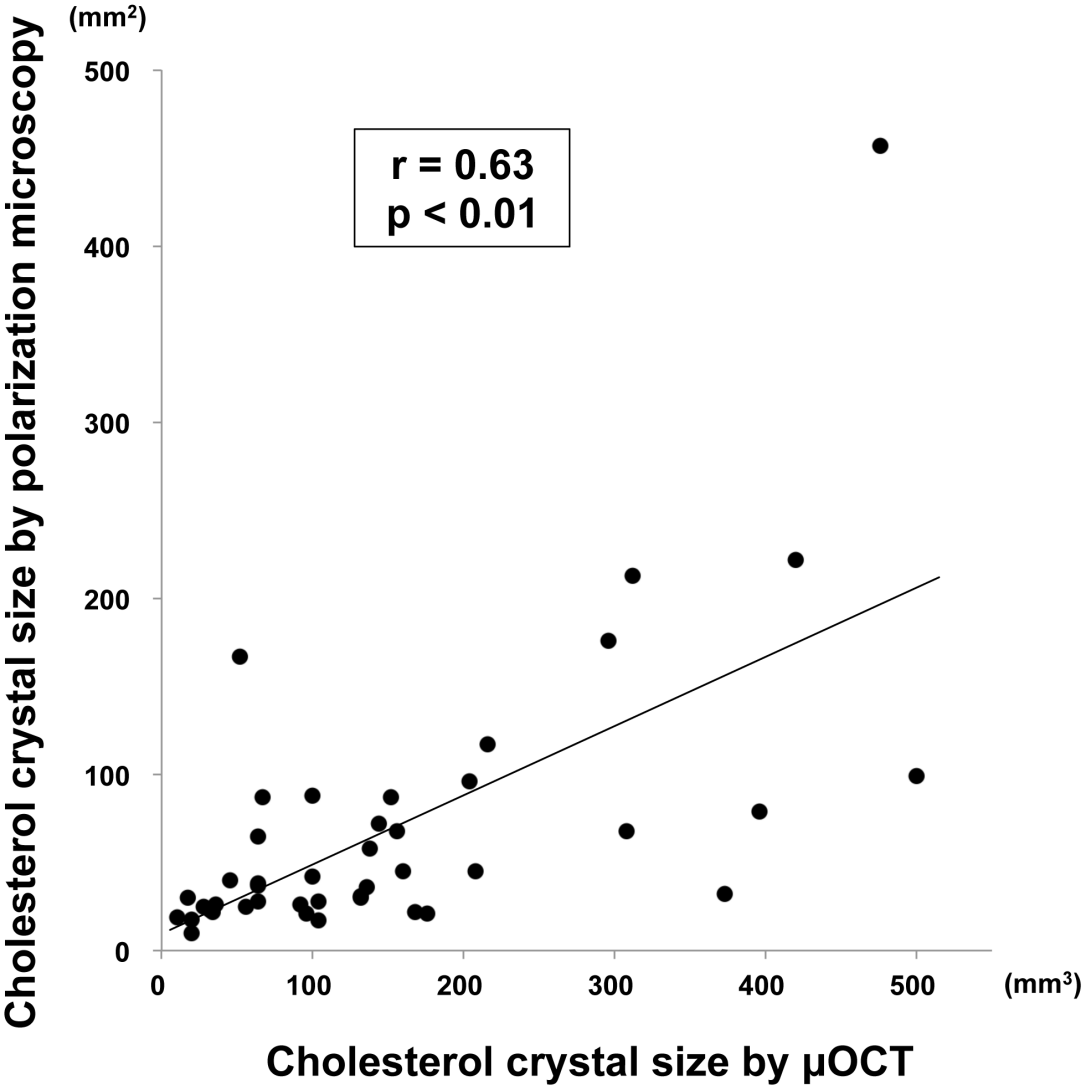
Correlation of cholesterol crystal size between µOCT and polarization microscopy. Cholesterol crystal sizes assessed by µOCT demonstrated significant correlations with those by polarization microscopy (r = 0.63 p<0.01).

We scanned 339 cultured macrophage cells and obtained matched images using µOCT, phase contrast, and polarization microscopy. A representative image set of macrophages containing cholesterol crystals is shown in Figure 3. On µOCT, the cholesterol crystal-containing macrophages demonstrated highly scattering inclusions within the cytoplasm matching the location of birefringent crystals visualized under polarization microscopy. Cholesterol crystal inclusions were not always evident by µOCT imaging however. Figure 4 illustrates such a case, in which the cholesterol crystals were detected by polarization microscopy but were not seen on the µOCT image. Using polarization microscopy as the gold standard, the sensitivity, specificity, positive predictive value (PPV) and negative predictive value (NPV) of µOCT for detecting cholesterol crystal within macrophages were measured to be 40.4%, 92.9%, 74.2% and 75.5%, respectively. Larger cholesterol crystals (≧100 mm^2^, assessed by polarization microscopy) were more accurately detected by µOCT than smaller crystals (<100 mm^2^) (52% vs 36%, p<0.05). Among macrophage cells in which µOCT could detect cholesterol crystals, the crystal volumes assessed by µOCT were significantly correlated with those by polarization microscopy (r = 0.63, p<0.01) (Figure 5).

### Tissue sample imaging

We scanned 45 cadaver human coronary artery segments containing macrophage cells by µOCT. The analysis results are summarized in Table 1. Because the µOCT image depth depends on sample properties, we applied 2D scale for scanning area.

**Table 1:** µOCT measured parameters from human coronary artery ex vivo.

Coronary lesion, n	45
Scanning area, µm^2^	1.80 [1.04–2.98]
Macrophage number, n	741
Macrophage density, /µm^2^	6.58 [1.41–16.9]
Long length, µm	35.57 [27.82–44.72]
Short length, µm	22.2 [17.26–27.16]
Volume, µm^3^	7543 [4117–13679]
With high scattering constituent, n	360 (49%)
Without high scattering constituent, n	381 (51%)

Data presented are median [interquartile range] or number count (%).

**Figure 6:**
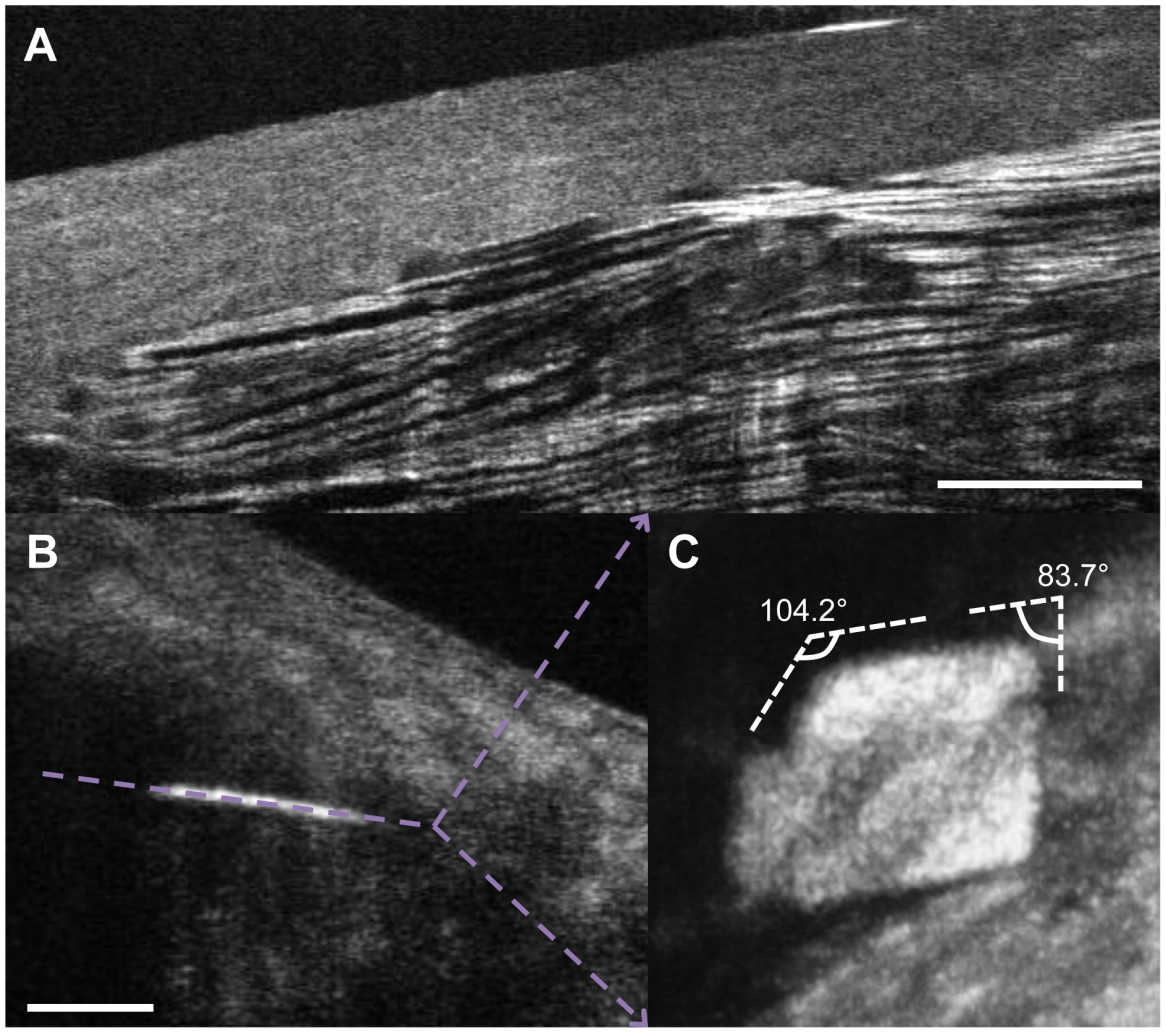
Representative images of cholesterol crystal in human tissue. The upper figure is a representative image of cholesterol crystal in human coronary artery, characterized by multiple intense reflections from its top and bottom surfaces (A). An en face image of a cholesterol crystal (B) is shown in panel (C). This transverse cut shows the typical angles associated with cholesterol monohydrate crystals of (83.7 (acute) and 104.2 (obtuse) degrees). Scale bars = 100 µm

µOCT clearly revealed extracellular cholesterol crystals within arterial tissue, which were characterized by intense reflections from its top and bottom surfaces (Figure 6). Transverse section of the µOCT images showed that these crystals were cholesterol monohydrate as typified by the average crystal angles of 76.68±5.83 (acute) and 105.58±6.31 (obtuse) degrees of 15 randomly selected samples, which were consistent with a previous report (79.15 and 100.85 degrees) [20].

**Figure 7:**
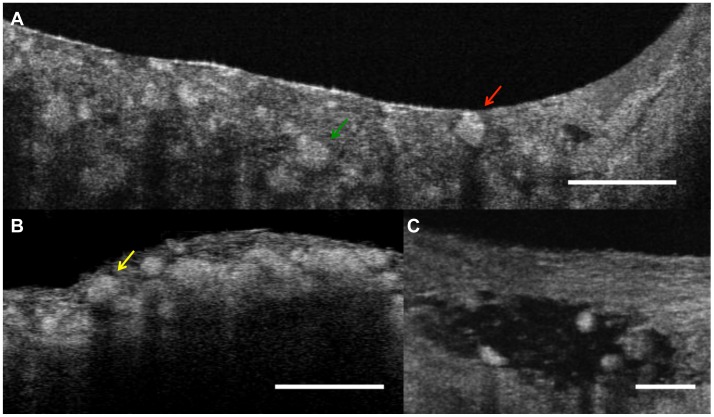
Macrophage cells in coronary artery. A. Macrophages (green arrow) seen by µOCT appear as highly scattering ellipsoidal structures that can be clearly distinguished from other coronary artery cellular and subcellular components. The macrophage on the right contains a highly scattering inclusion that is consistent with a cholesterol crystal (red arrow). B. Necrotic core fibroatheroma with macrophages (yellow arrow) infiltrating the cap. C. Some macrophages attenuated the OCT signal deep to the cells. Scale bars = 100 µm (A, B), 50 µm (C).

**Figure 8:**
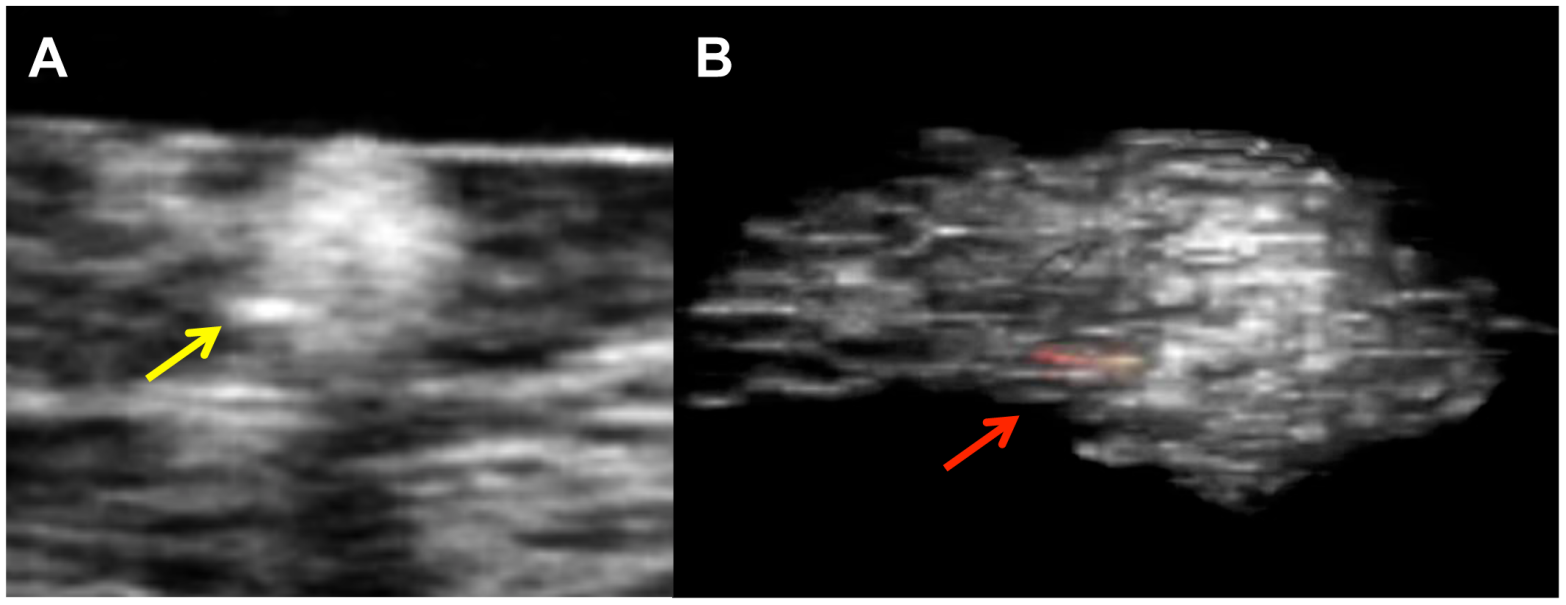
Macrophage cells with highly scattering constituents. A. Representative image of macrophage cells in human coronary artery contained highly scattering inclusions within their cytoplasm. B. Three-dimensional image of a macrophage showing a highly scattering inclusion (red-yellow color) within its cytoplasm (red arrow).

Macrophage volumes were readily quantified by µOCT. Macrophages in human tissue specimens were visualized as highly scattering, round or ellipsoidal structures within the coronary artery that were clearly delineated from other coronary artery components (Figure 7) [16]. We found 741 total macrophages, of which 49% contained highly scattering inclusions within their cytoplasm. Figure 8 shows one example of a macrophage containing a highly scattering inclusion that is also clearly visualized after 3D rendering the cell. This figure also shows that the pseudopods of the macrophage are oriented in the direction of the crystal, suggesting that this macrophage is “polarized” in the direction of the putative foreign body.

## Discussion and Conclusion

OCT imaging has proven to be a unique tool for high-resolution evaluation of the coronary artery [9], [12]. Crystalline cholesterol is one of the major coronary artery components that show the highest intensity signals on OCT image in human coronary artery [15], [22]. In our results, macrophages that contain cholesterol crystals demonstrated highly scattering inclusions in their cytoplasm, and the feasibility of µOCT to detect cholesterol crystals in macrophage cells quantitatively with high specificity *in vitro* was shown. Furthermore, we found some macrophages in fresh human cadaver coronary arteries demonstrated µOCT evidence of high intensity inclusions within their cytoplasm, consistent with the appearance of cholesterol crystals observed *in vitro*.

Macrophages play a key role in all phases of atherosclerosis. In particular, macrophages accumulate in vulnerable plaques prone to rupture, which causes an acute coronary event [23]. Macrophages and other plaque-related cells produce proteolytic enzymes that digest extracellular matrix and compromise the integrity of the fibrous cap. Therefore, in addition to assessment of cholesterol phagocytosis, quantitative assessment of macrophage cell distributions and sizes are also important for evaluating arterial inflammation. We have shown that µOCT is capable of morphological quantification of macrophage cells. This detailed information that is derived from µOCT could enable the further exploration for atherosclerosis beyond that possible with other existing imaging devices.

The present study has several limitations. We used synthetic cholesterol, which was artificially crystallized for macrophage cell cultures. These crystals might differ subtly from those crystallized in macrophages and human coronary artery *in vivo*. The detection sensitivity was low. Since light scattered from cholesterol crystals competes with scattering organelles also present in macrophages, intracellular contrast of the crystals was inconsistent. However, because cholesterol crystals are strongly birefringent, the addition of polarization sensitivity to the µOCT system is likely to improve detection of cholesterol crystals [24]. µOCT, as currently implemented using our bench top setup, is only appropriate for *ex vivo or in vitro* samples. When µOCT is conducted *in vivo,* issues such as motion artifacts will need to be ameliorated in order to obtain clear imaging of tissue microstructure. It can sometimes be difficult to confirm whether or not macrophages in plaque contain cholesterol crystals within their cytoplasm because the cholesterol crystal is soluble in the organic agents used in histology. Therefore, we don't have the golden standard for macrophage cells with cholesterol crystals in human plaques at current time and the diagnostic accuracy of µOCT for detecting those cells.

In conclusion, µOCT demonstrated the ability to assess cholesterol crystals in macrophage cells and to quantify macrophage size distributions. These results confirm that µOCT is a promising tool to visualize and quantify the interaction of macrophages and crystalline cholesterol, and underscores the potential beneficial impacts of *in vivo* µOCT imaging, including better understanding of coronary artery disease and evaluation of response to therapeutics that affect crystal-inflammasome interaction.
